# Evaluation of a mental health first aid workshop for healthcare professionals

**DOI:** 10.1192/bjo.2021.67

**Published:** 2021-06-18

**Authors:** Sara Abou Sherif, Sachin Patel

**Affiliations:** Hammersmith & Fulham Liaison Psychiatry, Charing Cross Hospital, Imperial College NHS Healthcare Trust, London UK

## Abstract

**Aims:**

Annually, 1 in 4 people in the UK will experience a mental health problem. Alongside the approach of increasing awareness of the issue amongst the general population, there is a drive to deliver training and education on the recognition and management of mental health crises. Limited resources exist to aid healthcare professionals in delivering mental health first aid (MHFA), with the vast majority focussing on lengthy training courses. Small group problem-based learning (PBL) is utilised widely in medical education and this modality offers advantages in deliverability, audience participation and experiential learning. Our aim was to deliver and explore the effectiveness of a PBL MHFA workshop to various healthcare professionals.

**Method:**

As part of an Emergency Medicine Mental Health Education day, we delivered four 30-minute PBL MHFA workshops. These involved an introduction to MHFA, followed by an interactive discussion of 4 mental health simulated cases, whereby participants anonymously answered a range of questions using the web-based platform Mentimeter. We devised a simple MHFA A,B,C,D,E acronym to bring structure to problem solving. Pre- and post-workshop questionnaires were used to assess outcomes using Likert scales to measure various aspects of MHFA (1 = strongly disagree and 5 = strongly agree). Statistical significance was calculated using T-Test with P < 0.05 defining statistical significance.

**Result:**

A total of 28 professionals attended the workshops, 20 (72%) completed both the pre and post workshop questionnaire. 19 (76%) were nurses (5 Registered Mental Health Nurses and 14 Registered General Nurses), 3 (12%) were doctors, 2 (8%) were HCA's and 1 was a policeman. 15 (75%) of the participants reported historically having had the need to deliver MHFA but only 3 (15%) had previously received training. After the workshop, participants reported significantly increased understanding [3.0 to 4.3 (p < 0.05)] and confidence in delivering MHFA [3.05 to 4.30 (p < 0.05)]. There was significantly improved confidence in assessing risk [3.03 to 4.05], calling for appropriate help [3.45 to 4.35] and de-escalation techniques [3.05 to 4.15].

**Conclusion:**

To our knowledge this is the first mini PBL-based MHFA workshop. We have demonstrated that the PBL workshop setup is an effective means to deliver training on MHFA. We recognise the importance of MHFA training reaching a larger audience and its potential value if incorporated into national healthcare training programmes and made available to the general public.

